# Expression of COX-1, COX-2, 5-LOX and $\hbox {CysLT}_2$ in nasal polyps and bronchial tissue of patients with aspirin exacerbated airway disease

**DOI:** 10.1186/s13223-019-0395-5

**Published:** 2019-12-26

**Authors:** Monique Vorsprach, Christoph Arens, Stephan Knipping, Dörte Jechorek, Sabine Stegemann-Koniszewski, Eva Lücke, Jens Schreiber

**Affiliations:** 1 Departmemt of Pneumology, University Hospital Magdeburg, Medical Faculty, Otto-von-Guericke-University, Leipziger Straße 44, 39120 Magdeburg, Germany; 2 Department of Otorhinolaryngology, Head- and Neck Surgery, University Hospital Magdeburg, Medical Faculty, Otto-von-Guericke-University, Leipziger Straße 44, 39120 Magdeburg, Germany; 3 Department of Otorhinolaryngology, Head- and Neck Surgery, Plastical Surgery, Dessau Medical Center, Martin-Luther-University Halle, Auenweg 38, 06847 Dessau, Germany; 4 Department of Pathology, University Hospital Magdeburg, Medical Faculty, Otto-von-Guericke-University, Leipziger Straße 44, 39120 Magdeburg, Germany

**Keywords:** Asthma, Nasal polyps, Aspirin exacerbated respiratory disease, Arachidonic acid, Cyclooxygenases, Lipoxygenases, Leukotrienes

## Abstract

**Background:**

Aspirin exacerbated respiratory disease (AERD) is a disease of the upper and lower airways. It is characterized by severe asthma, chronic sinusitis with nasal polyps (CRSwNP) and intolerance towards nonsteroidal analgesics (NSAR). Arachidonic acid (AA) metabolites play an important role in the pathogenesis of AERD. It is still unknown, whether metabolism of AA is comparable between the upper and lower airways as well as between patients with and without NSAR intolerance.

**Objective:**

We sought to analyze differences in the expression of cyclooxygenases type 1 and 2 (COX-1, COX-2), arachidonate 5-lipoxygenase (5-LOX) and cysteinyl leukotriene receptor type 2 ($\hbox {CysLT}_2$) in nasal polyps and the bronchial mucosa of patients with aspirin intolerant asthma (AIA, $n=23$) as compared to patients with aspirin tolerant asthma (ATA, $n=17$) and a control group with nasal polyps, but without asthma (NPwA, $n=15$).

**Methods:**

Tissue biopsies from nasal polyps and bronchial mucosa were obtained during surgical treatment of nasal polyps by endonasal functional endoscopic sinus surgery (FESS) under general anesthesia from intubated patients. Immunohistochemistry was used to analyze the expression of COX-1, COX-2, 5-LOX and $\hbox {CysLT}_2$ in nasal and bronchial mucosa. Categorization into the different patient groups was performed according to the patient history, clinical and laboratory data, pulmonary function and provocation tests, as well as allergy testing.

**Results:**

We observed a stronger expression of 5-LOX and $\hbox {CysLT}_2$ in submucosal glands of nasal and bronchial tissue compared to epithelial expression. The expression of COX-1 and COX-2 was stronger in epithelia compared to submucosal glands. There was a similar expression of the enzymes and $\hbox {CysLT}_2$ between upper and lower airways in all patient groups. We did not detect any significant differences between the patient groups.

**Conclusions:**

The AA-metabolizing enzymes and the $\hbox {CysLT}_2$ were expressed in a very similar way in different microscopic structures in samples of the upper and lower airways of individual patients. We did not detect differences between the patient groups indicating the pathogenetic role of AA metabolism in these disorders is independent of the presence of NSAR-intolerance.

## Background

Aspirin exacerbated respiratory disease (AERD; formerly “aspirin-induced asthma”) is a frequent disorder. A study published in 2014 points out, that there is a prevalence of 7% of AERD within asthma patients. Among patients with severe asthma it can even reach 14%. Furthermore, patients with chronic sinusitis with nasal polyps (CRSwNP) show a prevalence between 9 and 10% of AERD [1]. Performing aspirin provocation tests, it has been shown, that AERD is an underdiagnosed disorder [2]. AERD is usually characterized by upper and lower airway involvement. CRSwNP as well as bronchial asthma are hallmarks of this disorder. Typically, asthma deteriorates after exposure to aspirin or other nonsteroidal anti-inflammatory drugs (NSAID). Frequently asthma in AERD is nonatopic, severe and difficult to treat. Upper airway involvement has a significant impact on patients’ quality of life. The nasal condition is named chronical hyperplastic eosinophilic sinusitis (CHES) [3] and resembles bronchial asthma immunologically. Both in upper and lower airways Th-2 like immune responses are crucial, involving the cytokines IL-3, IL-4, IL-5, IL-13, eotaxin and GM-CSF [4–6] as well as activated eosinophils [7]. Nevertheless, only a part of patients with Th-2-mediated asthma suffer from intolerance to NSAID. These substances inhibit cyclooxygenase type 1 (COX-1), an enzyme which metabolizes arachidonic acid (AA) towards prostaglandins (PGs) and thromboxanes (TXs) [8, 9]. Following hydrolysis from the phospholipid layer, the AA metabolism follows two major pathways. On the one hand side leukotrienes (LTs) are being produced by 5-LOX and on the other hand side PGs and TXs through the COX pathway. There are two isotypes of the COX enzyme. While COX-1 is a ubiquitously occurring enzyme, COX-2 expression occurs after cytokine stimulation in inflamed tissue only [10]. The cysteinyl leukotrienes (Cys-LTs) are metabolites produced through the 5-LOX pathway leading to bronchoconstriction, vasodilatation, mucus production and recruitment of neutrophils und eosinophils in the lung through the corresponding receptors type 1 and 2 ($\hbox {CysLT}_2$) [10–14]. All these phenomena are hallmarks of severe asthma. It is assumed, that in AERD there is an imbalance in the metabolism of AA. Inhibition of COX-1 results in a reduction of PGs and a consecutive shift towards the LOX-pathway with increased production of LTs ($\hbox {LTC}_4$, LTD$_4$, LTE$_4$) which are regarded to play a pivotal role in the pathogenesis of asthma [10]. Furthermore, a role of prostaglandin E2 (PGE$_2$) in upper and lower respiratory tract involvement in AERD is assumed [15]. PGE$_2$ is a pleiotropic metabolite harboring anti-inflammatory, anti-fibrotic and immunerestrictive potential, that can at the same time also mediate proinflammatory responses [15–17].

Although there is good evidence for a pathogenetic role of AA-metabolism in respiratory diseases [10, 18], it is still unknown whether the relevant enzymes are expressed similarly in upper and lower airways. To the best of our knowledge, no studies have been reported in which AA-metabolizing enzymes were analyzed comparatively in nasal polyps and bronchial mucosa of the same subjects. Therefore, with regard to the “one airway, one disease” concept [19], we analyzed the immunoreactivity of COX-1, COX-2, 5-LOX and $\hbox {CysLT}_2$ in both nasal polyps and bronchial mucosa specimens from patients with aspirin intolerant asthma (AIA) in comparison to aspirin tolerant asthma patients (ATA) and controls with nasal polyps, but without asthma (NPwA).

## Methods

### Subjects and study design

We included patients, which were admitted for nasal polyposis surgery. These patients were divided into three groups depending on the presence of asthma with or without NSAID intolerance. Asthma was defined according to current guidelines based on the results of medical history with a standardized questionnaire (Asthma Control Test; ACT) [20] and pulmonary function tests including inhalative methacholine provocation tests. Standard skin prick testing defined atopy. Aspirin sensitivity was identified by inhalative aspirin provocation challenge in patients suffering from asthma. Patients of the control group suffered from CRSwNP with indication for surgery without a diagnosis of asthma. The study was approved by the Ethics Committee of the Faculty of Medicine of the Otto-von-Guericke-University Magdeburg and all patients gave written informed consent.

### Sample collection

Endonasal functional endoscopic sinus surgery (FESS) was performed in intubated and ventilated patients. Resected nasal polyps were preserved for further analysis. Fiberoptic bronchoscopy was performed via the endotracheal tube and 3–4 biopsies of the bronchial mucosa were obtained from the right main bronchus. The samples were preserved in 4% formalin until analysis. Automated dehydration was performed with ethylic alcohol (3 × 100%, 2 × 96% and 1 × 75%) and xylol, followed by embedding in paraffin. The specimens were cut into 3 $\mu$ m slices by a microtome (RM 2155, Leica Instruments GmbH, Nussloch, Germany) and standardized hematoxylin and eosin (H&E)-staining was performed for overview staining.

### Immunohistochemistry and image analysis

The immunohistochemical detection of 5-LOX, COX-1, COX-2 and $\hbox {CysLT}_2$ was performed on paraffin slices. After dehydration, dewaxing and doubled hydration of the slices, further procedures where performed, using standard automised conditions in a BENCHMARK® ULTRA Immunostainer (Ventana, Tucson, USA). After antigen demasking of the slices and blocking of endogenous biotin (iVIEW®-BLOCKER, Ventana), incubation with the primary and secondary antibody was performed. We used specific antibodies against 5-LOX (1:50; Abcam, Cambridge, UK), COX-1, COX-2 (both 1:50; Cayman Chemical, Ann Arbor, USA) and $\hbox {CysLT}_2$ (1:100; Abcam, Cambridge, UK) as previously described [21–23]. The color reaction was performed using iVIEW diamino-benzidine (iVIEW®-DAB) and the color enhancer iVIEW®-COPPER (both Ventana). Subsequently, hematoxylin staining was carried out. Before covering the slices with Canada-balm, dehydration using alcohol and xylol was performed. As a positive control, stomach mucosa was used. As a negative control the primary antibody was substituted by mouse or rabbit IgG antibodies (ab27479/ab27478, Abcam, Cambridge, UK). For the semiquantitative evaluation of the immunohistochemical reaction towards 5-LOX, $\hbox {CysLT}_2$, COX-1 und COX-2, light microcopy in 100-times to 400-times resolution (Eclipse E200, Nikon, Japan) was performed. The analysis of the cytoplasmic expression in epithelial cells of nasal and bronchial tissue was performed separately for the respiratory epithelium, the squamous epithelium and the local submucosal glands. In order to evaluate immunoreactivity, we used the immunoreactive score (IRS) of Remmele et al. [24, 25]. We focused on staining intensity (SI) and the frequency of immunopositive cells (percent positive; PP). The scale for SI included values from 0 to 3, whereas 0 implied no, 1 a low, 2 a moderate and 3 a strong staining. The scale for PP ranged from 1 to 10 in which the percentage of immunopositive cells out of the total epithelial cells was determined in 10% steps, e.g. 5 = 50% of the cells were immunopositive. In each specimen SI and PP was evaluated for 3 representative regions of each kind of epithelial layer to determine a mean value. From these values the immunoreactive score (IRS = SI x PP) was calculated. The preparations were fully examined. If one type of epithelium or mucosal glands were not detected, this was noted as a missing value in the statistical data collection.

### Statistical analysis

Data analysis was conducted using the software SPSS® (Statistical Packages for Social Sciences, Version 25 for Microsoft Windows®). For the statisticcal analysis of two groups a standard t-test for independent variables was performed. In this study we focused mainly on the comparison of three groups, for which ANOVA (analysis of variances) was performed by post hoc analysis (multiple testing, Tukey). $P< 0.05$ indicated statistically significant differences. In order to compare between the different tissue types within subjects, we used a mixed linear model. We determined the patient group characteristics and the tissue type as factors for the mixed model. Paired tissue comparisons were performed by Bonferroni correction.

## Results

### Clinical data and laboratory parameters

Clinical and demographic details as well as laboratory parameters of the study subjects are shown in Table 1.

**Table 1:** Clinical data and laboratory parameters of the study subjects

Group	AIA ($n=23$)	ATA ($n=17$)	NPwA ($n=15$)
Male/female (n)	11/12	12/4	11/4
Age in years, mean (range)	48.7 (18–70)	51.2 (40–67)	43.2 (19–68)
Asthmatic patients, n	23	17	0
Atopic patients, n (%)	8 (34%)	8 (47%)	0
Number of previous FESS operations; mean (range)	2 (1–8)	1.4 (1–6)	1.38 (1–2)
Absolute eosinophilic blood counts (Gpt/l); mean (range)	0.486 (0.134–0.872)	0.263 (0.079–0.458)	0.116 (0.1–0.15)
Eosinophilic infiltrations in nasal polyps, n (%)	18 (78.3%)	14 (82.3%)	11 (73.3%)
Eosinophilic infiltrations in bronchial mucosa, n (%)	5 (21.7%)	3 (17.6%)	1 (6.6%)

The table shows the parameters as means and range

AIA patients showed increased counts of eosinophilic granulocytes with a statistically significant difference between AIA and NPwA ($p=0.001$) and between ATA as compared to NPwA patients ($p=0.009$), respectively (Table 1). Atopy, based on skin prick test results, was present in 34% of AIA and 47% of ATA, but none of the NPwA patients. The differences between the two groups of asthmatics did not reach statistical significance (Table 1).

### Histology of nasal polyps and bronchial tissue

In nasal specimens from ethmoidal, maxillary, frontal and sphenoid sinuses respiratory mucosa with goblet cells was detected histologically. Some samples showed subepithelial infiltrations with lymphocytes, plasma cells and eosinophils. The mucosa frequently showed an edematous appearance in all groups. Bronchial biopsies ranged from 0.2 to 0.6 cm in size. We detected bronchial ciliated epithe-lium with occasional goblet cells in all specimens. Some samples showed squamous epithelial metaplasia. We observed an extensive inflammatory infiltration in some samples, in others only a few lymphocytes. We also observed local mucosal glands and bronchial smooth muscle cells. These observations were not specific for particular patient groups. Infiltrations of eosinophilic granulocytes were found in nasal polyp samples of all groups. In bronchial tissue we in general detected less eosinophilic infiltrations, most likely due to the small size of the biopsy samples (Table 1).

### Expression of 5-LOX

**Fig. 1:**
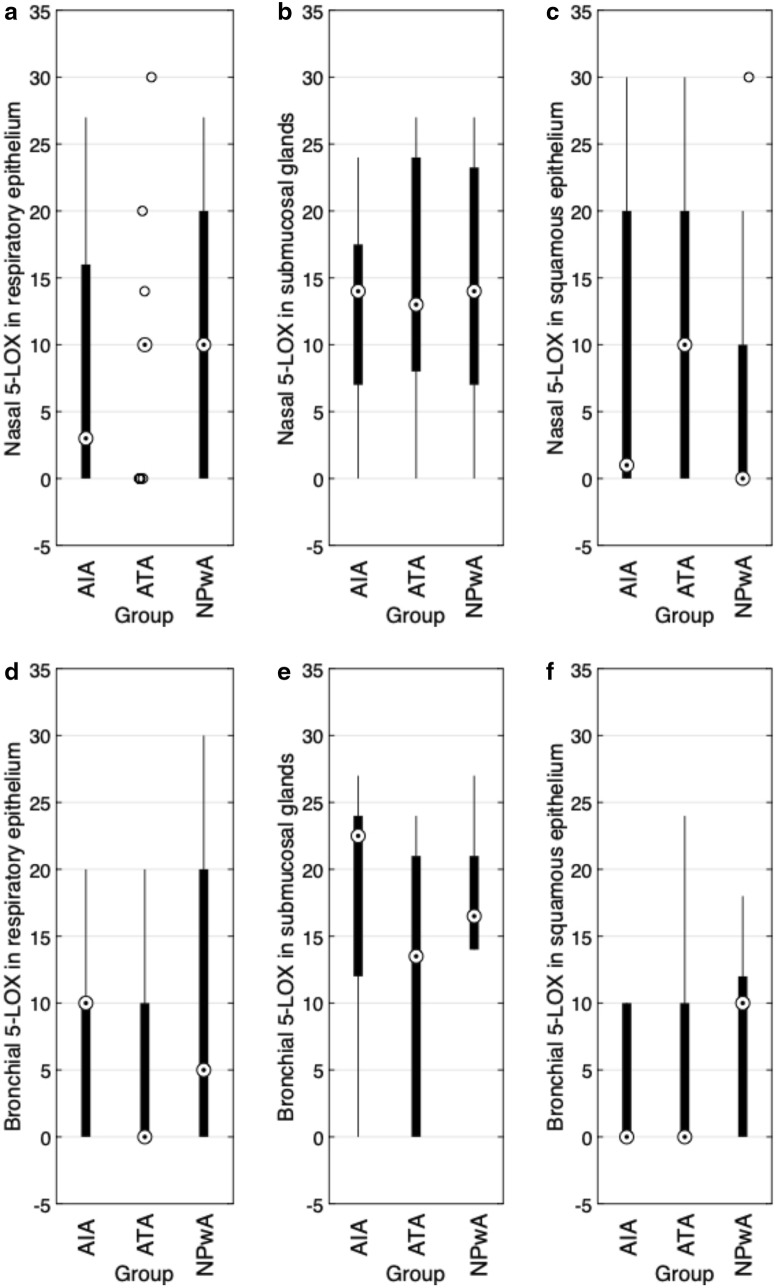
5-LOX immunoreactivity (IRS). Results of the staining of nasal (**a**–**c**) and bronchial (**d**–**f**) samples for 5-LOX are shown as boxplot diagrams. The lower and upper boundaries indicate the 25% and 75% quartile, respectively. Minimum and maximum values are indicated as whiskers and the dot represents the group median. Outliers were plotted as individual points. **a** Nasal respiratory epithelium (AIA: $n=22$, ATA: $n=16$, NPwA: $n=15$), **b** nasal submucosal glands (AIA: $n=22$, ATA: $n=16$, NPwA: $n=14$) and **c** nasal squamous epithelium (AIA: $n=18$, ATA: $n=12$, NPwA: $n=12$), without significant differences in patient group comparisons. **d** Bronchial respiratory epithelium (AIA: $n=15$, ATA: $n=14$, NPwA: $n=8$), **e** bronchial submucosal glands (AIA: $n=11$, ATA: $n=9$, NPwA: $n=6$) and **f** bronchial squamous epithelium (AIA: $n=10$, ATA: $n=12$, NPwA: $n=5$), without significant differences in patient group comparison

Nasal immunoreactivity for 5-LOX did not show significant differences between groups (Fig. 1a–c). The highest expression of 5-LOX was observed in submucosal glands, with a statistically significant difference between ″ glands ($p=0.038$), (Table 2, Fig. 4a). Similar observations were made comparing submucosal glands with squamous epithelium, these differences however did not reach statistical significance ($p=0.094$) (Table 2). In bronchial tissue, we observed a medium 5-LOX expression in submucosal glands and a low expression in the bronchial epithelial layers (Fig. 1d–f) with a highly significant difference between glands and epithelium (each $p<0.001$) (Table 2). Comparing patient groups, we detected a stronger expression of 5-LOX in the respiratory epithelial layer of controls as compared to asthmatic patients, however not reaching statistical significance (Fig. 1d).

**Table 2:** Comparison of the expression of 5-LOX, COX-1, COX-2 and $\hbox {CysLT}_2$ between the respiratory epithelium, the submucosal glands and the squamous epithelium

	RE; mean (SE)	SG mean (SE)	SEP mean (SE)	*p*-value RE vs. SG	*p*-value SEP vs. SG	*p*-value RE vs. SEP
5-LOX nasal polyps	9.645 (1.197)	13.588 (1.241)	9.42 (1.59)	0.038	0.094	NS
5-LOX bronchial tissue	7.266 (1.458)	15.901 (1.778)	6.915 (1.516)	0.001	0.001	NS
COX-1 nasal polyps	13.287 (1.82)	10.24 (1.42)	13.784 (1.774)	0.001	0.003	NS
COX-1 bronchial tissue	10.611 (1.912)	5.103 (1.959)	11.83 (2.077)	0.028	0.003	NS
COX-2- nasal polyps	21.245 (1.335)	13.763 (0.978)	20.959 (1.494)	< 0.001	< 0.001	NS
COX-2 bronchial tissue	14.47 (1.898)	7.406 (1.581)	13.794 (1.947)	0.002	0.011	NS
$\hbox {CysLT}_2$ nasal polyps	3.041 (0.895)	18.851 (1.125)	3.078 (0.895)	< 0.001	< 0.001	NS
$\hbox {CysLT}_2$ bronchial tissue	1.938 (0.851)	13.969 (1.841)	1.936 (0.581)	< 0.001	< 0.001	NS

General comparison of the mean immunoreactive score (IRS) of the respiratory epithelium (RE), submucosal glands (SG) and squamous epithelium (SEP), irrespective of the patients’ groups, using the mixed linear statistical model. Mean IRS are indicated +/− standard error (SE). *NS* nonsignificant

### Expression of COX-1

**Fig. 2:**
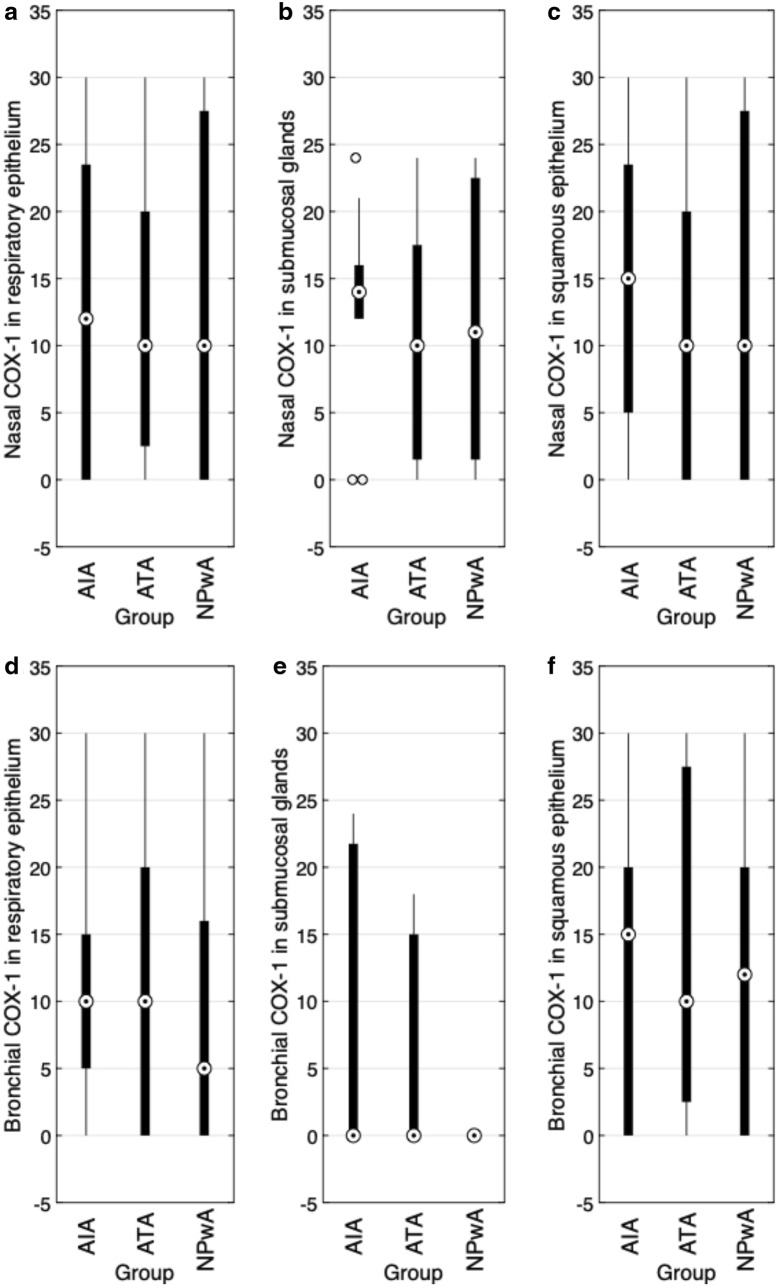
COX-1 immunoreactivity (IRS). Results of the staining of nasal (**a**–**c**) and bronchial (**d**–**f**) samples for COX-1 are shown as boxplot diagrams. The lower and upper boundaries indicate the 25% and 75% quartile, respectively. Minimum and maximum values are indicated as whiskers and the dot represents the group median. Outliers were plotted as individual points. **a** Nasal respiratory epithelium (AIA: $n=15$, ATA: $n=12$, NPwA: $n=15$), **b** nasal submucosal glands (AIA: $n=12$, ATA: $n=14$, NPwA: $n=11$) and **c** nasal squamous epithelium (AIA: $n=15$, ATA: $n=13$, NPwA: $n=6$), without significant differences in patient group comparisons. **d** Bronchial respiratory epithelium (AIA: $n=13$, ATA: $n=14$, NPwA: $n=8$), **e** bronchial submucosal glands (AIA: $n=5$, ATA: $n=5$, NP: $n=3$) and **f** bronchial squamous epithelium (AIA: $n=11$, ATA: $n=10$, NPwA: $n=6$), without significant differences in patient group comparisons

The expression of COX-1 in polypoid nasal tissue showed no statistically significant differences between the patient groups. We detected a medium COX-1 expression in all investigated epithelial layers and submucosal glands (Figs. 2a–c, 4b). As for 5-LOX, also the analysis of COX-1 expression revealed a significant difference between submucosal glands and the respiratory ($p=0.001$) as well as the squamous epithelium ($p=0.003$), with the lowest IRS in submucosal glands (Table 2). While medium COX-1 immunoreactivity was detected in the bronchial tissue of asthmatic patients in the submucosal glands and epithelia, there was no COX-1 expression in the submucosal glands of controls (Fig. 2d–f). Furthermore, we observed a significant difference between low bronchial COX-1 expression in submucosal glands and a stronger IRS in the respiratory ($p=0.0028$) and squamous epithelium ($p=0.003$), respectively, with the highest expression in the squamous epithelium (Table 2).

### Expression of COX-2

**Fig. 3:**
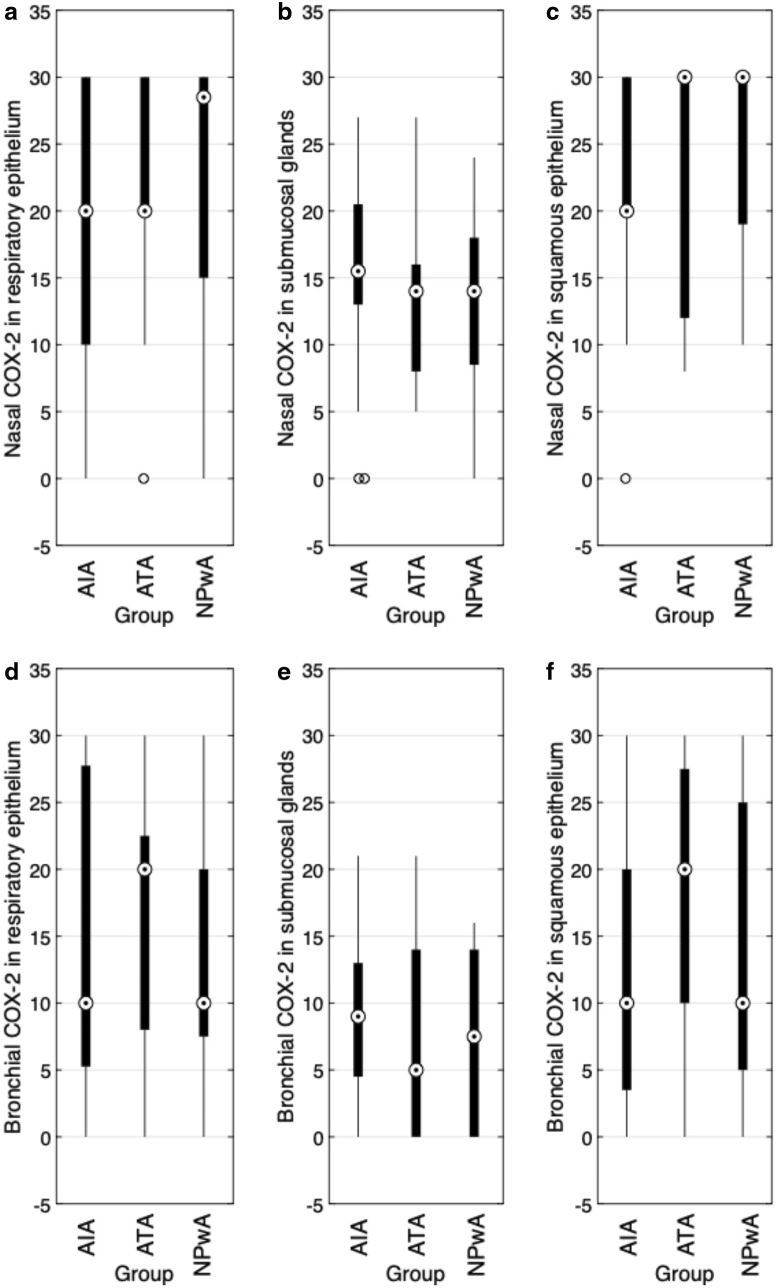
COX-2 immunoreactivity (IRS). Results of the staining of nasal (**a**–**c**) and bronchial (**d**–**f**) samples for COX-2 are shown as boxplot diagrams. The lower and upper boundaries indicate the 25% and 75% quartile, respectively. Minimum and maximum values are indicated as whiskers and the dot represents the group median. Outliers were plotted as individual points. **a** Nasal respiratory epithelium (AIA: $n=23$, ATA: $n=17$, NPwA: $n=15$), **b** nasal submucosal glands glands (AIA: $n=19$, ATA: $n=16$, NP: $n=15$) and **c**) nasal squamous epithelium (AIA: $n=13$, ATA: $n=13$, NPwA: $n=12$), without significant differences in patient group comparisons. **d** Bronchial respiratory epithelium (AIA: $n=14$, ATA: $n=13$, NPwA: $n=8$), **e** bronchial submucosal glands (AIA: $n=7$, ATA: $n=8$, NPwA: $n=5$) and **f** bronchial squamous epithelium (AIA: $n=13$, ATA: $n=11$, NPwA: $n=7$), without significant differences in patient group comparisons

**Fig. 4:**
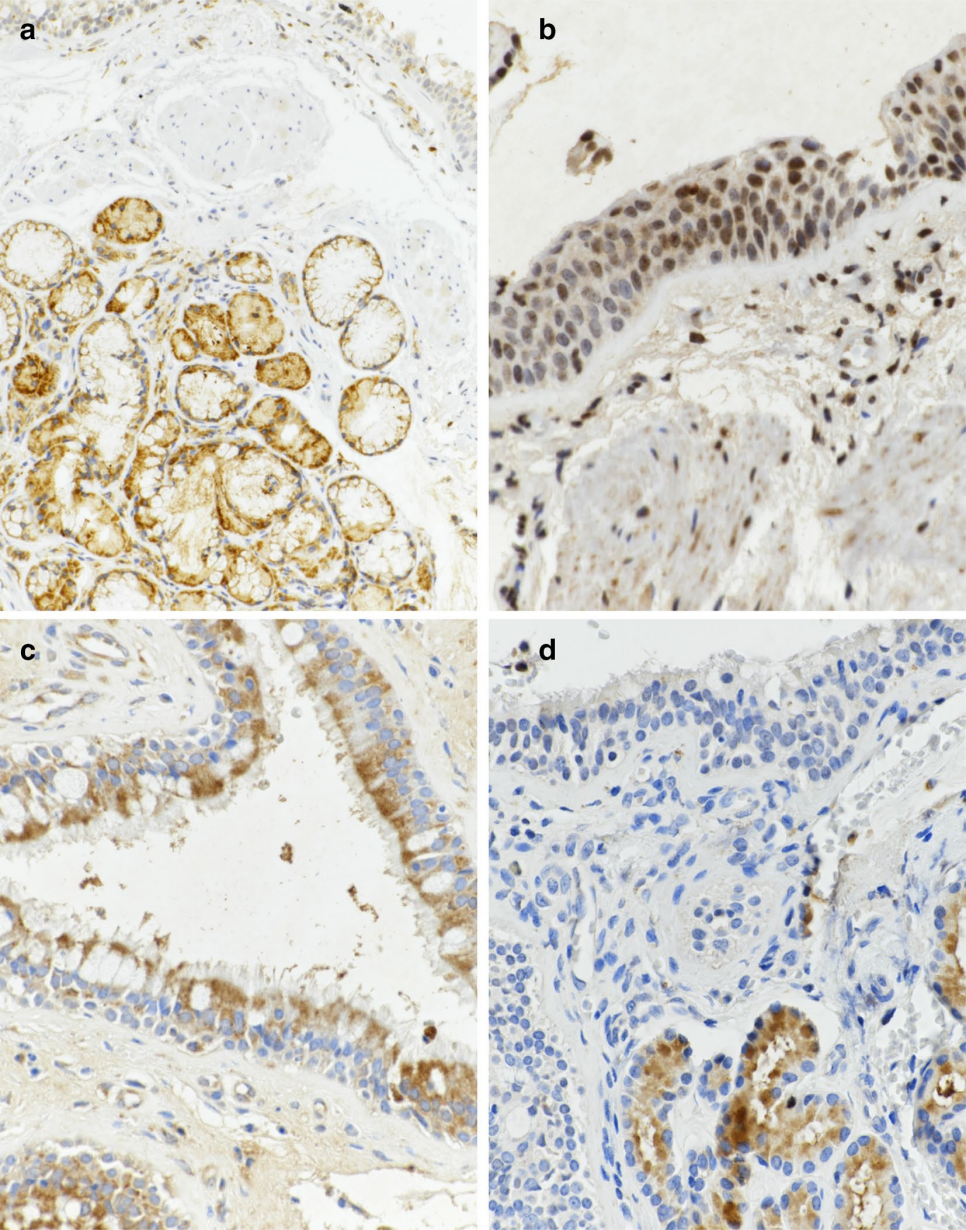
Representative sections of specimens immunostained for **a** 5-LOX (respiratory epithelium and submucosal glands of nasal polyp), **b** COX-1 (bronchial mucosa with squamous metaplasia), **c** COX-2 (respiratory epithelium of nasal polyp) and **d** $\hbox {CysLT}_2$ receptor (respiratory epithelium and submucosal glands of bronchial mucosa) from AIA patients. Brown staining represents protein expression. X 400

Analyzing the expression of the inducible COX-2 in nasal polyp tissue, we observed strong immunoreactivity within the epithelial layers with IRS values up to the maximum score of 30 (Figs. 3a–c, 4c). Again, we detected highly significant differences between submucosal glands and each type of epithelium ($p<0.001$) (Table 2). As for COX-1, COX-2 expression was lowest in the submucosal glands as compared to the respiratory as well as squamous epithelium. However, we did not detect any remarkable patient group differences (Fig. 3a–c). Regarding bronchial COX-2 expression, we observed a significantly increased mean expression in epithelia (respiratory: $p=0.002$ and squamous: $p=0.011$) as compared to submucosal glands without any patient group differences (Fig. 3d–f).

### Expression of $\hbox {CysLT}_2$

**Fig. 5:**
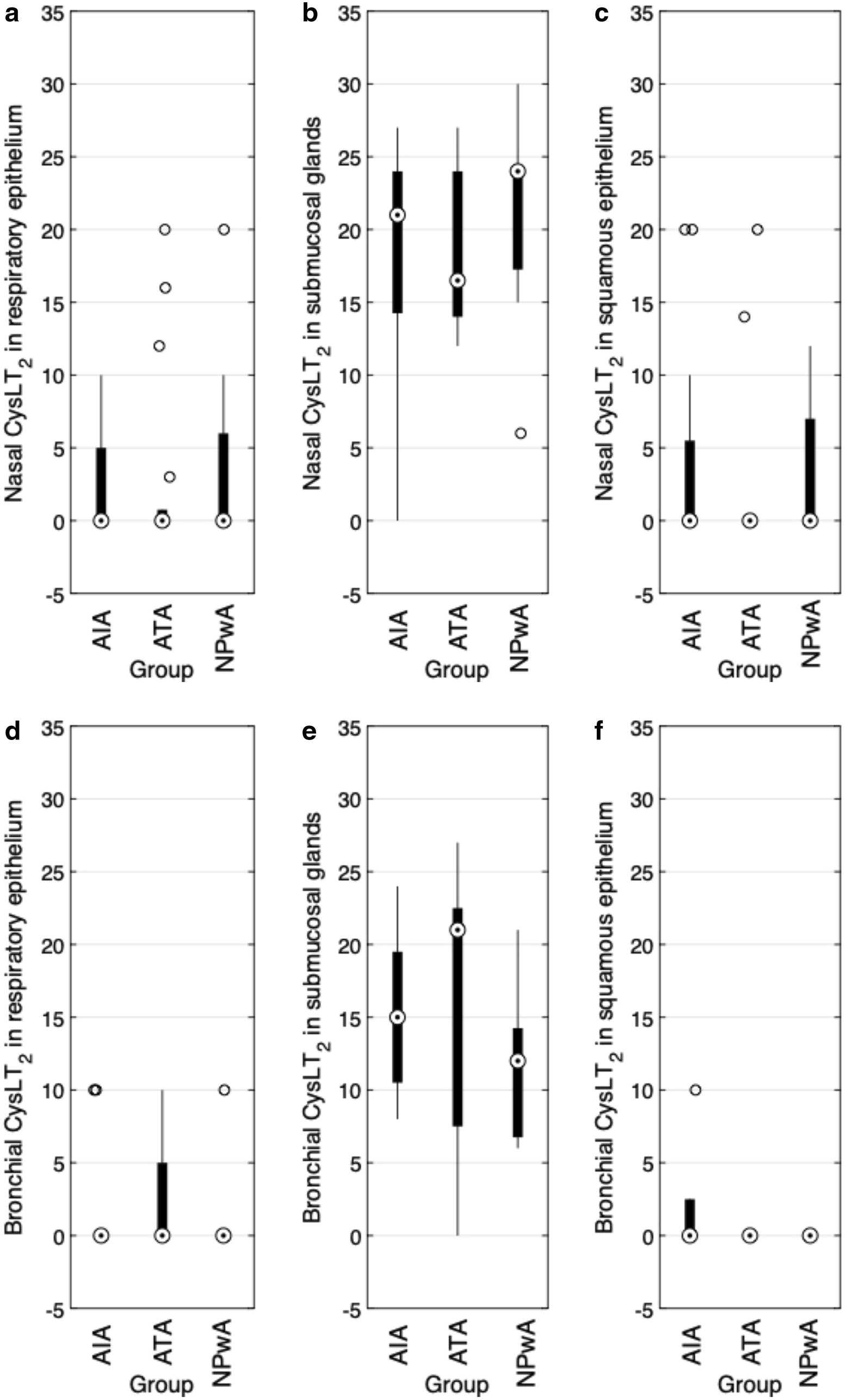
$\hbox {CysLT}_2$ immunoreactivity (IRS). Results of the staining of nasal (**a**–**c**) and bronchial (**d**–**f**) samples for $\hbox {CysLT}_2$ are shown as boxplot diagrams. The lower and upper boundaries indicate the 25% and 75% quartile, respectively. Minimum and maximum values are indicated as whiskers and the dot represents the group median. Outliers were plotted as individual points. **a** Nasal respiratory epithelium (AIA: $n=19$, ATA: $n=14$, NPwA: $n=14$), **b** nasal submucosal glands (AIA: $n=15$, ATA: $n=15$, NPwA: $n=12$) and **c** nasal squamous epithelium (AIA: $n=15$, ATA: $n=14$, NPwA: $n=14$), without significant differences in patient group comparisons. **d** Bronchial respiratory epithelium (AIA: $n=13$, ATA: $n=8$, NPwA: $n=5$), **e** bronchial submucosal glands (AIA: $n=7$, ATA: $n=6$, NPwA: $n=4$) and **f** bronchial squamous epithelium (AIA: $n=5$, ATA: $n=1$, NPwA: $n=3$), without significant differences in patient group comparisons

Analyzing $\hbox {CysLT}_2$ immunoreactivity in nasal tissue, we detected a medium expression in submucosal glands that was clearly and significantly reduced in the epithelia (each $p<0.001$) (Table 2, Fig. 4d). “ between the patient groups (Fig. 5a–c). In the respiratory epithelium of bronchial tissue, we detected $\hbox {CysLT}_2$ expression only in a few subjects, in the squamous epithelium only in one subject (Fig. 5c–f). Epithelial cells mainly showed immunoreactivity in the apical part of the goblet cells. A medium expression was found in the analyzed submucosal glands and comparing means, we detected highly significant differences between submucosal glands and the epithelia (each $p<0.001$) (Table 2).

## Discussion

In this study we analyzed the expression of COX-1, COX-2, 5-LOX and $\hbox {CysLT}_2$ in both nasal polyps and bronchial mucosa from patients with AIA in comparison to ATA patients and non-asthmatic controls with nasal polyps (NPwA). In contrast to other studies, we focused on analyzing immunoreactivity in both nasal and bronchial tissue of the same subjects.

Clinically, we detected 34 % atopic patients within the AIA group by standard skin-prick testing. This is in contrast to the common assumption that AIA patients are mostly nonatopic asthmatics [26]. At the same time, this finding is well in line with the AIANE study by Szczeklik et al. [2] where likewise 34 % of AIA patients revealed atopy by skin-prick test. In our study blood counts of eosinophils were significantly increased between AIA and ATA and controls ($p=0.001$), as well as between ATA and controls ($p=0.001$), respectively. This is in accordance with several published observations, indicating an important pathogenetic role of these cells. Thus, Yamaguchi et al. [27] analyzed blood and nasal sinus eosinophil counts in 30 ATA und AIA patients. While the eosinophilic count in ATA sinuses did not differ as compared to controls, AIA patients showed significantly increased ($p<0.05$) eosinophilic concentrations in ethmoidal cells. Furthermore, a positive correlation was found between blood and nasal sinus eosinophil counts and concentrations in ethmoidal cells ($r=0.5249$; $p<0.0001$). Therefore, our data regarding significantly increased blood eosinophil counts in AIA are in line with these previous reports, but this cell population does not discriminate between AIA and ATA patients.

Regarding the expression of LOX-5, we observed a stronger 5-LOX expression in submucosal glands of nasal polyps and bronchial tissue as compared to the epithelial layers. Overall, we detected medium 5-LOX expression without significant differences between the patient groups. In line with our results, Owens et al. [23] report medium 5-LOX expression in submucosal glands in their immunohistochemical analysis, but with a significant difference between ATA and NP patients. Adamjee et al. [22] detected a three-fold increased 5-LOX expression in AIA as compared to ATA patients in their analysis of epithelial cells of nasal polyps. Altogether, currently the literature towards 5-LOX expression in the airways is rather limited and future studies will be needed to address these discrepancies and to clarify the role of 5-LOX expression in AIA.

Also, with regard to the nasal and bronchial expression of COX-1, we did not detect group differences. In the studies of Gosepath et al. [28] and Demoly et al. [29], a distinctive COX-1 expression in patients with nasal polyps and CRS was observed, also without significant differences [28, 29]. Cowburn et al. [30] detected similar results in bronchial tissue of AIA patients. Mullol et al. [31] found a lower COX-1 expression in nasal polyps of ATA patients without increase after cytokine exposure. In a study of Westergren et al. [32] in patients with allergic rhinitis, increased epithelial COX-1 expression was detected. This was also associated with increased numbers of intraepithelial mast cells. Owens et al. [23] analyzed the nasal expression of cyclooxygenases and lipoxygenases in AIA, ATA and control patients. They observed a rather increased epithelial expression of COX-1 in AIA und ATA patients as compared to controls. As in our study, the glandular COX-1 expression showed no patient group differences. Those reports suggest that the expression of COX-1 possibly plays a certain role in the formation of nasal polyps. However, the results of our study do not confirm this hypothesis and therefore suggest that the contribution of COX-1 expression to pathophysiology is only limited. Of note COX-1 is a constitutive expressed enzyme, possibly explaining the similar expression in all analyzed patients’ groups.

Analyzing the expression of the inducible enzyme COX-2, we also did not observe significant patient group differences. Even though not statistically significant, the epithelial expression of COX-2 in nasal polyps of patients with AIA appeared lower as compared to ATA or NP patients (Fig. 3). Comparing the mean expression, we generally observed a lower COX-2 expression in submucosal glands with no patient group difference (Table 2). Demoly et al. [29] also detected no differences of epithelial cyclooxygenase expression in patients suffering of CRS. In line with our results of slightly reduced COX-2 protein expression in nasal polyps of AIA patients, Pujols et al. [33] showed in a real-time PCR approach analyzing the dynamics of COX-2 expression in nasal tissue of AIA and ATA patients, that the baseline concentrations of COX-2 mRNA in nasal polyps were significantly reduced as compared to the nasal mucosa in both groups. While COX-2 mRNA expression in the nasal mucosa did not change after 1 h at room temperature, it increased significantly in ATA nasal polyps but not in in AIA patients. Those results were related to the imbalanced arachidonic acid metabolism. In contrast, Yun et al. [34] detected COX-2 expression in submucosal glands, cytoplasm, mucosa, endothelial cells and vascular walls of nasal polyps. Owen et al. [23] detected an increased expression of COX-2 both in epithelial layers and submucosal glands, without group differences. In another study of Gosepath et al. [28], a lower COX-2 epithelial expression in nasal polyps versus CRS patients was found as compared to inflamed nasal mucosa. This possibly suggests, that COX-2 is an inducible enzyme in inflammatory tissue. In general, the available data on COX-2 expression are limited and as for 5-LOX and COX-1, further studies, also addressing enzyme activity, will be needed to comprehensively clarify their role for pathophysiology and as a marker in AIA. Interestingly, also patients in the NPwA group showed a relevant expression of the AA-metabolizing enzymes in the bronchial mucosa, even in the absence of clinical manifest asthma. This indicates that in these patients latent, clinically inapparent inflammatory reactions may occur in lower airways. We also did not detect significant group differences regarding the expression of the $\hbox {CysLT}_2$ receptor. Cys-LTs play an important role in the respiratory tract in the pathophysiology of AERD. An excessive cysteinyl- leukotriene production is characteristic for this disorder not only reflected by increased basal urine levels, but also after aspirin provocation [35–38]. In order to gain knowledge about the role of leucotriene in AERD, we did not analyze the enzyme expression of $\hbox {LTC}_4$ synthase, but the expression of the $\hbox {CysLT}_2$ receptor. In mammals there are two types of Cys-LT receptors, which function as a classical G-proteins [39]. The Cys-LT receptor type 1, which usually occurs in bronchial muscle cells, but also in macrophages and mast cells, has a higher affinity towards LTD$_4$ and the pharmaceutical leukotriene antagonists montelukast, zafirlukast and pranlukast, which play an important role in standard asthma therapy [14]. We chose to analyze $\hbox {CysLT}_2$, because it binds equally to the bronchoconstrictors $\hbox {LTC}_4$ and LTD$_4$, but also with a higher affinity than the CysLT$_1$ receptor. In addition to its expression in myeloid cells and smooth muscle tissue, $\hbox {CysLT}_2$ is also found in endothelial cells, cardiac Purkinje cells and in brain cells [12, 40]. In a study of Adamjee et al. [22], AIA patients showed four times more $\hbox {LTC}_4$ positive cells than ATA patients. Additionally, 5-LOX was expressed three times as strong in AIA than in ATA, without detecting any differences in the expression of COX-1 or COX-2. They also detected a five times increased blood eosinophilic count in AIA and a positive correlation between $\hbox {LTC}_4$ positive eosinophils and mucosal eosinophils [22]. Similar results were described by the same group in bronchial tissues of AIA, ATA and NP patients. Here, a five times higher expression of $\hbox {LTC}_4$ synthase in AIA was shown, as compared to ATA patients and 18 times higher towards controls [30]. Those results led to the assumption that a higher expression of $\hbox {LTC}_4$ synthase in AIA patients in mucosal eosinophils might be correlated to the occurrence of AERD in the upper and lower airways. Corrigan et al. [21] found a higher expression of $\hbox {CysLT}_2$ in submucosal glands of paranasal sinuses of ATA patients as compared to controls. The detection of similar $\hbox {CysLT}_2$ expression levels in the epithelium and submucosal glands within nasal and bronchial samples of AIA, ATA and NP patients in our study however suggests only a limited role for $\hbox {CysLT}_2$ expression in these cells in AERD.

Aspirin desensitization therapy and leukotriene receptor antagonists are treatment strategies, as they interfere with the arachidonic acid metabolism, although aspirin desensitization is more effective in most patients. Our results show similarities in arachidonic acid metabolism between nasal polyps and lower airways. This might be a pathophysiologic basis for the efficacy of the treatment in upper, as well as in lower airways.

## Conclusion

The expression of the AA-metabolizing enzymes and the CysLT-receptor 2 was very similar in the upper and lower airways of individual patients, indicating a role of the AA pathway in both manifestations of these disorders. Therefore, these results support the “one airway, one disease” concept [19].

We detected, in both nasal and bronchial tissue, a stronger expression of 5-LOX and $\hbox {CysLT}_2$ in submucosal glands as compared to the epithelial expression indicating a relevance the LOX-pathway of AA-metabolism in these mucus producing structures. The nasal and bronchial epithelial expression of constitutive COX-1 and inducible COX-2 was stronger as compared to submucosal glands in both, indicating, that prostaglandin pathway of AA may play a more important role in epithelial cells. We did, however, not detect significant differences between the patient groups, which is in line with the assumption, that AA-metabolism is activated in a similar way in AIA and ATA with comparable disease severity. These enzyme expressions could be detected even in the lower airways of patients without bronchial asthma, indicating clinically inapparent changes. Our findings suggest that the postulated imbalance in AA metabolism possibly does not take place at the level of enzyme expression. This is partly a contradiction to the current literature. Comparing different studies, differences in mRNA abundance versus protein expression, which we analyzed in our study, need to be taken into account. Future studies, possibly applying multiple detection methods and larger patient groups will be needed to further increase our pathophysiological understanding of the alterations in AA metabolism in AERD and the key players in these.

## Data Availability

The datasets used and analyzed during the current study are available from the corresponding author on reasonable request.

## Abbreviations

ACT: asthma control test
AERD: aspirin exacerbated respiratory disease
AIA: analgesic intolerant bronchial asthma
ATA: analgesic tolerant bronchial asthma
CHES: chronical hyperplastic eosinophilic sinusitis
CRSwNP: chronic sinusitis with nasal polyps
COX-1: cyclooxygenase 1
COX-2: cyclooxygenase 2
CRS: chronical rhinosinusitis
CysLTs: cysteinyl leukotrienes
$\hbox {CysLT}_2$: cysteinyl leukotriene receptor type 2
FESS: functional endoscopic sinus surgery
GM-CSF: granulocyte macrophage colony-stimulating factor
H[MYAMPE]: hematoxylin and eosin staining
IgG: immunglobulin G
Il-3: interleukin 3
Il-4: interleukin 4
Il-5: interleukin 5
Il-13: interleukin 13
IRS: immune reactive score
5-LOX: 5-lipoxygenase
$$\hbox {LTC}_4$$: cysteinyl leukotriene C4
$\hbox {LTC}_4$S: cysteinyl leukotriene C4 synthase
$$\hbox {LTD}_4$$: cysteinyl leukotriene D4
$$\hbox {LTE}_4$$: cysteinyl leukotriene E4
NPwA: nasal polyps without asthma
NSAID: nonsteroidal anti-inflammatory drug
PCR: polymerase chain reaction
PGs: prostaglandins
$$\hbox {PGE}_2$$: prostaglandin E2
PP: count of immunopositively cells
SS: staining intensity
$\hbox {Th}_2$: type 2 T helper cell
TXs: thromboxans
